# Comparative efficacy and safety of antiplatelet or anticoagulant therapy in patients with chronic coronary syndromes after percutaneous coronary intervention: A network meta-analysis of randomized controlled trials

**DOI:** 10.3389/fphar.2022.992376

**Published:** 2022-09-30

**Authors:** Yaowang Lin, Zhigang Cai, Shaohong Dong, Huadong Liu, Xinli Pang, Qiuling Chen, Jie Yuan, Qingshan Geng

**Affiliations:** ^1^ Department of Cardiology, Shenzhen People’s Hospital (The Second Clinical Medical College, Jinan University; The First Affiliated Hospital, Southern University of Science and Technology), Cardiovascular Minimally Invasive Medical Engineering Technology Research and Development Center, Shenzhen Key Medical Disciplin (SZXK003), Shenzhen, Guangdong, China; ^2^ Institution of Shenzhen Hospital, Guangzhou University of Chinese Medicine (Futian), Shenzhen, China; ^3^ Department of Pharmacy, Shenzhen People’s Hospital, Shenzhen, Guangdong, China; ^4^ Department of Geriatrics, Shenzhen People’s Hospital, Shenzhen, Guangdong, China

**Keywords:** antiplatelet therapy, chronic coronary syndromes, percutaneous coronary intervention, randomized control trials, geriatric disease, anticoagulant

## Abstract

Aimed to evaluate and compare the interactive effects of different antiplatelet or anticoagulation strategies in patients with chronic coronary syndromes (CCS) after percutaneous coronary intervention (PCI). Randomized controlled trials comparing different antiplatelet or anticoagulant strategies in patients with CCS after PCI were included. The primary outcomes were major adverse cardiovascular event (MACE), mortality, ischemic and bleeding events. Compared to aspirin alone, addition of prasugrel or ticagrelor to aspirin resulted in lower risk of myocardial infarction (MI) [odds ratio (OR): 0.38 (95% confidence interval 0.38–0.62); 0.810–0.84 (0.69–0.98)] and any stroke [0.56 (0.42–0.75)] at the expense of increased risk of major bleeding [1.79 (1.34–2.39); 2.08–2.38 (1.56–3.28)], whereas, clopidogrel monotherapy reduced the risk of any stroke, major bleeding, and intracranial bleeding. On subgroup analysis, compared with aspirin alone, addition of prasugrel resulted in lower MACE [0.72 (0.60–0.86)], MI [0.48 (0.38–0.62)], and stent thrombosis [0.29 (0.09–0.91)], whereas, addition of rivaroxaban 2.5 mg resulted in lower risk of MACE [0.72 (0.60–0.87)], cardiac death [0.71 (0.52–0.98)] and any stroke [0.65 (0.45–0.95)], but not reduced MI. Both prasugrel and rivaroxaban 2.5 mg increased major bleeding [1.79 (1.34–2.39); 1.72 (1.33–2.22)]. Clopidogrel monotherapy was associated with lower MACE [0.72 (0.58–0.90)], any stroke [0.42 (0.24–0.73)], and major bleeding [0.62 (0.40–0.96)]. Adding prasugrel or ticagrelor led to a reduced incidence of MI and prasugrel was also found to reduce the risk of MACE and stent thrombosis in CCS patients with low risk of bleeding after PCI. Clopidogrel monotherapy has advantage in reducing MACE, stroke, and major bleeding events in CCS patients at high risk of bleeding after PCI.

**Systematic Review Registration:** https://clinicaltrials.gov/, PROSPERO Identifier: CRD 42021291050.

## Introduction

Coronary artery disease (CAD) is a pathological process characterized by the formation of atherosclerotic plaques followed by their rupture, ulceration, or erosion (Libby and Theroux, 2005; Asada et al., 2020). Plaque rupture activates platelet aggregation and the coagulation cascade, which leads to acute coronary thrombosis, resulting in acute coronary syndrome (ACS) (Frangogiannis, 2015). Accordingly, antiplatelet and anticoagulant therapy has been recommended as a cornerstone treatment for CAD (Valgimigli et al., 2017).

Maintenance therapy with a single antiplatelet agent is the standard approach for secondary prevention of atherosclerotic cardiovascular events in patients with chronic coronary syndromes (CCS) (Knuuti et al., 2019). Aspirin, a cyclooxygenase pathway inhibitor, which reduces the formation of thromboxane A2 and inhibits platelet aggregation, is predominantly recommended as the standard-of-care monotherapy in patients with CCS (Godley and Hernandez-Vila, 2016). In 2017, the European Society of Cardiology recommended a combination of aspirin and ticagrelor (60 mg twice a day) for CCS patients with risk of ischemia (Ibanez et al., 2017). Similarly, the 2020 European Society of Cardiology update recommended the addition of a second antithrombotic agent (clopidogrel, prasugrel, or low-dose rivaroxaban) to aspirin for extended long-term secondary prevention in patients at high risk of ischemia and low risk of bleeding (Collet et al., 2020).

However, there is no clear consensus on the optimal post-percutaneous coronary intervention (PCI) antithrombotic strategy for CCS patients with respect to either replacement of aspirin with other antiplatelet agents or addition of a P2Y12 inhibitor or a low-dose anticoagulant to aspirin. Therefore, we conducted a network meta-analysis to compare the antithrombotic drugs with aspirin and assess their interactive effect on major adverse cardiovascular events (MACE), mortality, and ischemic and bleeding events in CCS after PCI.

## Materials and methods

### Search strategy

The present study was performed following the Cochrane Collaboration guidelines. Relevant articles published before 30 March 2022 were searched in online biomedical databases (PubMed and Clinical Trials. gov) and Cochrane Central Register (Supplementary Data Sheet S1; Supplementary Tables S1–S3). The keywords included “antiplatelet therapy,” “anticoagulant therapy,” “chronic coronary syndromes,” “stable coronary artery disease (SCAD),” and “randomized control trials (RCTs).” After elimination of duplicates using the EndNote software, two investigators (YL And QC) independently screened the titles and abstracts of the remaining articles using pre-defined criteria.

### Eligibility criteria

The inclusion criteria for studies were: 1) study design: randomized controlled trial; 2) study population: patients diagnosed with CCS after PCI; 3) intervention group received oral antiplatelet therapy and/or anticoagulant therapy; patients in the control group received aspirin or placebo in addition to aspirin; 4) outcomes: MACEs, mortality, ischemic events, and bleeding events; 5) language of publication: English.

### Study outcomes

The primary outcome was MACE. The secondary outcomes included myocardial infarction (MI), all-cause death, cardiac death, any stroke, major bleeding, fatal bleeding, intracranial bleeding, stent thrombosis, and any revascularization in patients with CCS after PCI.

### Data extraction and quality assessment

After independent screening of the titles and abstracts of relevant papers by two authors (YL and QC), the final decision on the inclusion of a study was made by consensus. Next, data were extracted from the full-text articles using standardized tables (including study design, interventions, endpoints, and follow-up data) and then checked independently. Any disagreements between the two authors were resolved by consensus or by consulting a third author (JY).

The risk of bias in the included studies was assessed using the Cochrane Collaboration tool; publication bias was assessed by visual inspection of Begg’s funnel plot; and the indirectness, imprecision, heterogeneity, and inconsistency of the included RCTs were assessed using network meta-analysis (CINeMA) framework (Papakonstantinou et al., 2020).

### Statistical analysis

STATA software, version 14.0 (Stata Corp, United States) was used for statistical analyses. Combined odds ratios (ORs) with 95% confidence intervals (CI) were calculated for the primary and secondary outcomes. Rankogram plotting was performed on the surface under the cumulative ranking (SUCRA) curve to provide a hierarchy of different treatments. Heterogeneity among the studies was assessed using the *I* ^*2*^ statistic. In case of significant heterogeneity (*I* ^*2*^ > 50%), subgroup analysis was performed to investigate heterogenity. Subgroup analyses were planned on the basis of factors identified *a priori* as potential sources of heterogeneity. *p-*values < 0.05 were considered indicative of statistical significance.

## Results

### Characteristics of included studies

**FIGURE 1:**
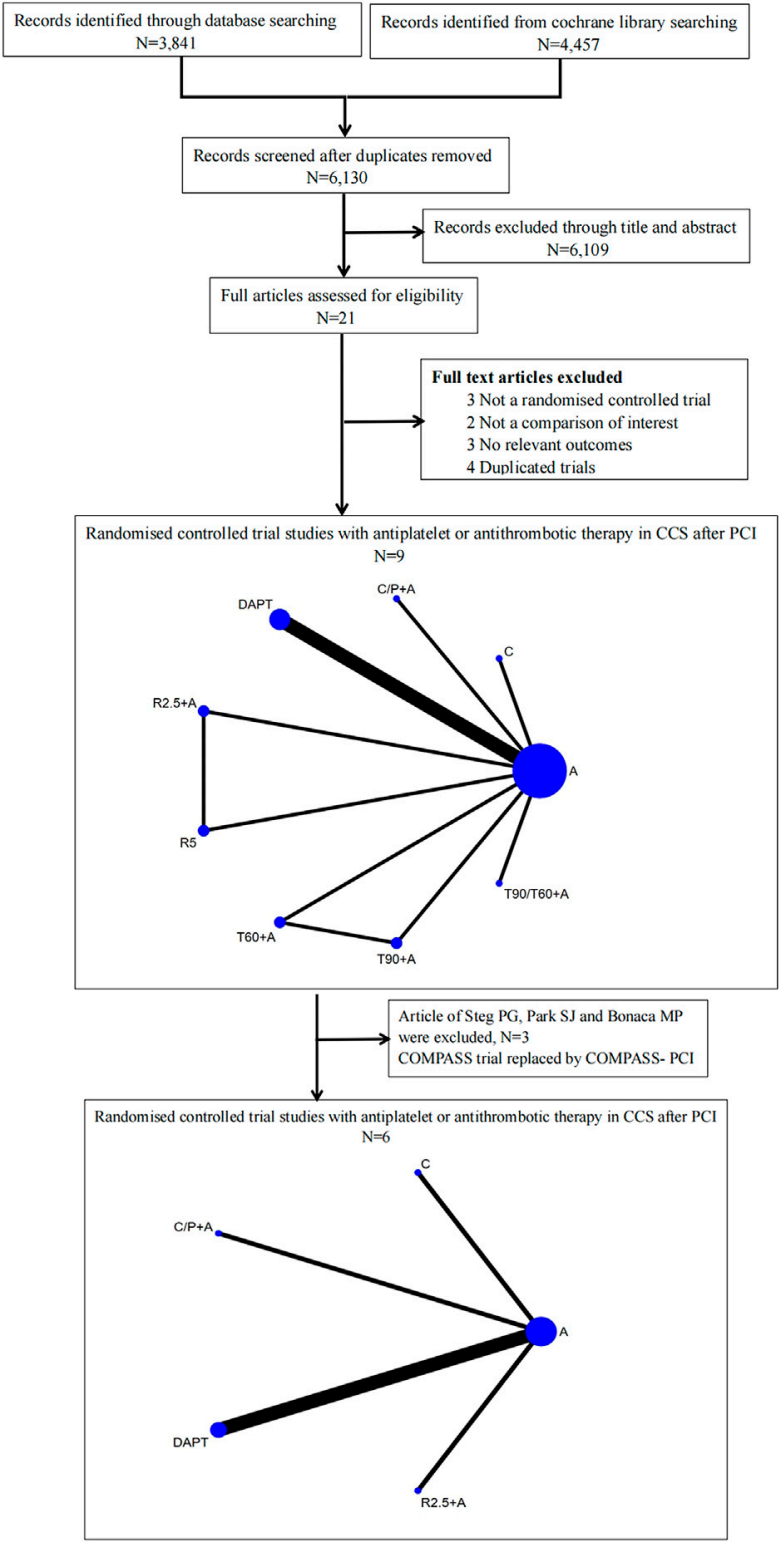
Flow diagram illustrating the study selection process and drug strategies in the Network. A, aspirin; DAPT, aspirin + clopidogrel; C/P + A, clopidogrel/prasugrel plus aspirin; T90 + A, ticagrelor 90 mg twice a day plus aspirin; T60, ticagrelor 60 mg twice a day plus aspirin; T90/60 + A, ticagrelor 90 mg/60 mg twice a day plus aspirin; R2.5 + A, rivaroxaban 2.5 mg twice a day plus aspirin; R5, rivaroxaban 5 mg twice a day; C, clopidogrel.

Out of the 8,298 articles retrieved on database search, 2,168 duplicate publications and 6,109 articles that did not qualify the inclusion criteria were excluded. In addition, 12 articles were excluded as these were not RCTs, duplicate trials, or no relevant endpoint data were reported. Eventually, nine RCTs (Figure 1) were included in this meta-analysis (Park et al., 2010; Collet et al., 2014; Lee et al., 2014; Mauri et al., 2014; Bonaca et al., 2015; Helft et al., 2016; Connolly et al., 2018; Steg et al., 2019; Koo et al., 2021). Among of these studies, aspirin and clopidogrel were used in five studies (Park et al., 2010; Collet et al., 2014; Lee et al., 2014; Mauri et al., 2014; Helft et al., 2016), aspirin and ticagrelor 90 mg b.i.d. in two studies (Bonaca et al., 2015; Steg et al., 2019), aspirin and rivaroxaban 2.5 mg b.i.d or 5 mg qd were used in one study (Connolly et al., 2018), and single clopidogrel was used in one study (Koo et al., 2021). The major bleeding was defined as TIMI in seven studies (Collet et al., 2014; Lee et al., 2014; Mauri et al., 2014; Helft et al., 2016; Connolly et al., 2018; Steg et al., 2019) and BARC type ≥ 3 in two studiers (Bonaca et al., 2015; Koo et al., 2021). A total of 91,115 patients were randomized to drug intervention group [*n* = 54,035, aspirin + clopidogrel (*n* = 5,218), aspirin + clopidogrel (*n* = 3,263)/prasugrel (*n* = 1,757), aspirin + ticagrelor 90 mg b.i.d (*n* = 7,050), aspirin + ticagrelor 60 mg b.i.d (*n* = 7,045), aspirin + ticagrelor 90 mg or 60 mg b.i.d (*n* = 9,619), aspirin + rivaroxaban 2.5 mg b.i.d (*n* = 8,313), rivaroxaban 5 mg (*n* = 8,250) and clopidogrel monotherapy (*n* = 2,710)] versus aspirin or aspirin plus placebo group (*n* = 37,080). The duration of follow-up ranged from 18 to 44 months. All studies were parallel RCTs, among which, four involved double-blinding, while five were open-label studies (Table 1).

**TABLE 1:** Baseline characteristics of the dTRA and TRA groups.

Included study	Year	Design	Participants	PCI	Total	Intervention group	Control group	MACE definition	MACE	All cause death
Park SJ, Korea	2010	REAL-LATE and ZEST-LATE trials	CAD with PCI > 12 months	100%	2,791	A+C (n = 1357)	A (n = 1344)	MI, stroke, or death from cardiac cause	28/1357 vs. 15/1344	20/1357 vs. 13/1344
Collet JP, France	2014	Multicentre, open-label, randomized trial (ARCTIC-Interruption)	CAD with PCI > 12 months	100%	1,259	A+C (91%)/P (9%) (n = 635)	A/P (8%) (n = 624)	Death, MI, stent thrombosis, stroke, or urgent revascularisation	24/635 vs. 27/624	7/635 vs. 9/624
Lee CW, Korea	2014	Multicentre, open-label, randomized trial (DES LATE)	CAD with PCI > 12 months	100%	5,045	A+C (n = 2531)	A (n = 2514)	Cardiac death, MI, or stroke	61/2531 vs. 57/2514	46/2531 vs. 32/2514
Mauri L, United States	2014	Multicentre, open-label, randomized trial (DAPT Study)	CAD with PCI > 12 months	100%	9,991	A+C (65%)/P (35%) (n = 5020)	A+placebo (n = 4941)	Death, MI or stroke	211/5020 vs. 285/4941	98/5020 vs. 74/4941
Bonaca MP, United States	2015	Randomized double-blind trial (PEGASUS-TIMI 54)	Myocardial infarction 1 to 3 years earlier	83.02%	21,162	A+T 90 (n = 7050)	A+placebo (n = 7067)	Cardiac death, MI, or stroke	493/7050 vs. 578/7067	326/7050 vs. 326/7067
				83.46%		A+T60 (n = 7045)			487/7045 vs. 578/7067	289/7045 vs. 326/7067
Helft G, France	2016	Multicentre, open-label, randomized trial (OPTIDUAL)	CAD with PCI > 12 months	100%	1,385	A+C (n = 695)	A (n = 690)	All-cause mortality, MI, stroke, or major bleeding	40/695 vs. 52/690	16/695 vs. 24/690
Connolly SJ, Canada	2018	Multicentre, double-blind, randomized, placebo-controlled (COMPASS)	CCS or with PAD	60%	24,824	A+R2.5 (n = 8313)	A (n = 8261)	Cardiac death, MI, or stroke	347/8313 vs. 460/8261	262/8313 vs. 339/8261
				60%		R5 (n = 8250)			411/8250 vs. 460/8261	316/8250 vs. 339/8261
Steg PG, USA	2019	Double-blind randomized trial (THEMIS)	CCS with diabetes without MI	79.8%	19,220	A+T 90/60 (n = 9619)	A+placebo (n = 9601)	Cardiac death, MI, or stroke	736/9619 vs. 818/9601	579/9619 vs. 592/9601
Bainey KR, Canada	2020	Double-blind randomized trial (COMPASS PCI)	CAD with PCI > 12 months	100%	9,862	A+R2.5 (n = 4963)	A (n = 4899)		201/4963 vs. 270/4899	125/4963 vs. 170/4899
Koo BK, Korea	2021	Multicentre, open-label, randomized trial (HOST-EXAM)	CAD with PCI > 12 months	100%	5438	C (n = 2710)	A (n = 2728)	All-cause death, MI, stroke, readmission due to ACS, and major bleeding	152/2710 vs. 207/2728	51/2710 vs. 36/2728

Cardiovascular death	Myocardial infarction	Any Stroke	Stent thrombosis	Any revascularisation	Bleeding definition	Major bleeding	Fatal bleeding	Intracranial bleeding	Follow-up
13/1357 vs. 8/1344	10/1357 vs. 7/1344	9/1357 vs. 4/1344	5/1357 vs. 4/1344	36/1357 vs. 26/1344	TIMI	3/1357 vs. 1/1344	0/1357 vs. 0/1344	—	24 months
—	9/635 vs. 9/624	6/635 vs. 4/624	0/635 vs. 3/624	8/635 vs. 9/624	TIMI	0/635 vs. 0/624	0/635 vs. 0/624	—	17 months
28/2531 vs. 19/2514	19/2531 vs. 27/2514	21/2531 vs. 21/2514	7/2531 vs. 11/2514	81/2531 vs. 65/2514	TIMI	34/2531 vs. 24/2514	1/2531 vs. 4/2514	5/2531 vs. 3/2514	24 months
45/5020 vs. 47/4941	99/5020 vs. 198/4941	37/5020 vs. 43/4941	19/5020 vs. 65/4941	—	BARC type ≥ 3	129/4713 vs. 72/4650	7/4713 vs. 4/4650	13/4713 vs. 9/4650	18 months
182/7050 vs. 210/7067	275/7050 vs. 338/7067	100/7050 vs. 122/7067	—	—	TIMI	127/6988 vs. 54/6996	6/6988 vs. 12/6996	29/6988 vs. 23/6996	36 months
174/7045 vs. 210/7067	285/7045 vs. 338/7067	91/7045 vs. 122/7067	—	—	—	115/6958 vs. 54/6996	11/6958 vs. 12/6996	28/6958 vs. 23/6996	—
10/695 vs. 14/690	11/695 vs. 16/690	5/695 vs. 7/690	6/695 vs. 1/690	35/695 vs. 35/690	TIMI	4/695 vs. 4/690	1/695 vs. 0/690	1/695 vs. 2/690	44 months
139/8313 vs. 184/8261	169/8313 vs. 195/8261	74/8313 vs. 130/8261	50/8313 vs. 46/8261	530/8313 vs. 553/8261	TIMI	263/8313 vs. 158/8261	14/8313 vs. 9/8261	26/8313 vs. 23/8261	36 months
175/8250 vs. 184/8261	176/8250 vs. 195/8261	105/8250 vs. 130/8261	50/8250 vs. 46/8261	527/8250 vs. 553/8261	—	236/8250 vs. 158/8261	12/8250 vs. 9/8261	43/8250 vs. 23/8261	—
364/9619 vs. 357/9601	274/9619 vs. 328/9601	180/9619 vs. 221/9601	—	828/9619 vs. 879/9601	TIMI	206/9562 vs. 100/9531	17/9562 vs. 10/9531	70/9562 vs. 46/9531	39.9 months
66/4963 vs. 91/4899	107/4963 vs. 134/4899	46/4963 vs. 69/4899	—	—	TIMI	165/4963 vs. 96/4899	7/4963 vs. 2/4899	17/4963 vs. 13/4899	36 months
19/2710 vs. 14/2728	18/2710 vs. 28/2728	18/2710 vs. 43/2728	10/2710 vs. 16/2728	56/2710 vs. 69/2728	BARC type ≥ 3	33/2710 vs. 53/2728	—	4/2710 vs. 17/2728	24 months

### Assessment of risk of bias, heterogeneity, and publication bias

Quality assessment of the included studies was performed using the Cochrane Collaboration tool in Review Manager 5.3. Each entry had a high risk of selection bias, detection bias, and reporting bias, respectively. Six studies had a high risk of performance bias. The remaining studies had a low risk of bias (Supplementary Data Sheet S2; Supplementary Figure S4). The funnel plot was asymmetrical indicated publication bias with different treatment effects in smaller studies and heterogeneity (*I* ^*2*^ *= 71.9*%; Supplementary Data Sheet S2; Supplementary Figure S5A). Subgroup analyses were conducted to explore by prespecified subgroup analysis (*I* ^*2*^ *= 0%*; Supplementary Data Sheet S2; Supplementary Figure S5B).

### Primary endpoint (major adverse cardiovascular event) and secondary endpoint of patients with chronic coronary syndromes after percutaneous coronary intervention

**FIGURE 2:**
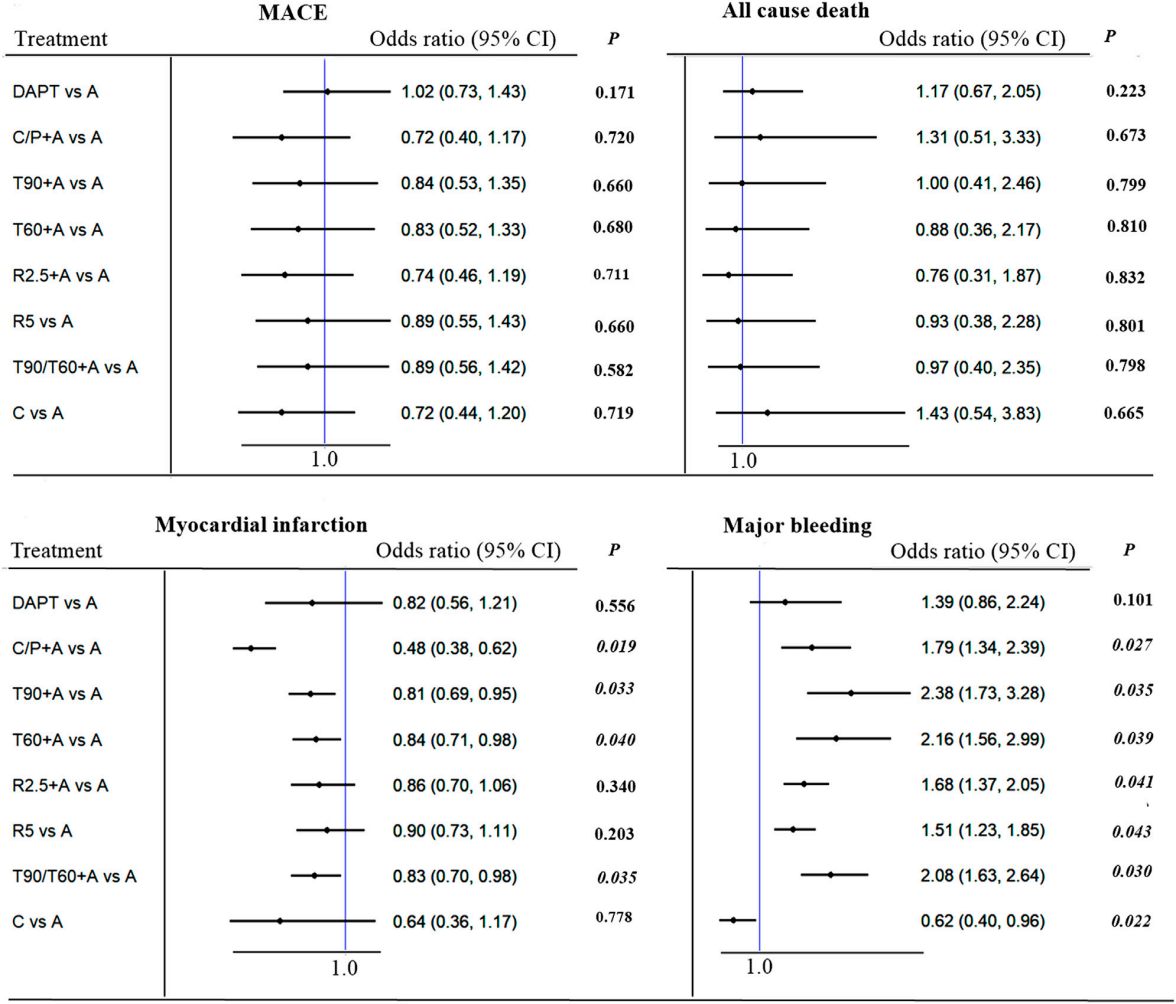
MACE (primary outcome), myocardial infarction, all cause death and major bleeding in patients with CCS after PCI: Forest plot (estimates as hazard ratio). Direct comparison from studies.

**FIGURE 3:**
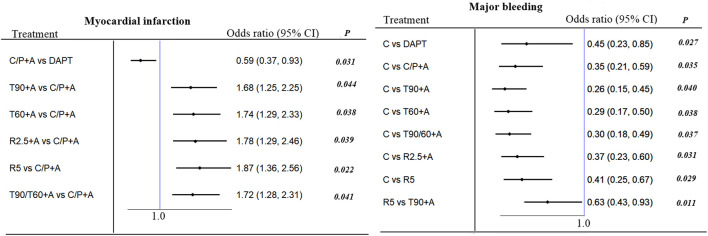
MACE, myocardial infarction, all cause death and major bleeding in patients with CCS after PCI: Forest plot (estimates as hazard ratio). Show significant difference between-group from indirect comparison of network.

No significant difference was observed between all antithrombotic treatment strategies with respect to primary endpoint of MACE. Compared to aspirin alone, adding prasugrel (not clopidogrel monotherapy) or ticagrelor resulted in lower MI [(0.38 (0.38–0.62), *p* = 0.019, number needed to treat (NNT) = 49; 0.810–0.84 (0.69–0.98), *p* = 0.033 to 0.040, NNT = 114 to 137] at the expense of increased major bleeding [(1.79 (1.34–2.39), *p* = 0.027, NNT = 84; 2.08–2.38 (1.56–3.28), *p* = 0.020 to 0.035; NNT = 95 to 114, Figure 2]. On indirect comparison, adding prasugrel was superior to ticagrelor, low-dose rivaroxaban, and clopidogrel monotherapy; therefore, prasugrel (at a maintenance dose of 10 mg daily in patients weighing >60 kg and a dose of 5 mg daily in patients weighing <60 kg) may be the optimal additional antithrombotic agent in reducing MI (Figure 3) in patients with CCS after PCI. From SUCRA rankogram plots, adding prasugrel was the best treatment strategy for both reducing MACE and MI in CCS after PCI (Supplementary Data Sheet S2; Supplementary Figure S1).

Clopidogrel monotherapy was predominately associated with a lower risk of any stroke [0.42 (0.24–0.73), *p* = 0.021, NNT = 109], major bleeding [0.62 (0.40–0.96), *p* = 0.040, NNT = 139], and intracranial bleeding [0.24 (0.08–0.70), *p* = 0.029, NNT = 213], whereas, rivaroxaban 2.5 mg plus aspirin reduced the incidence of any stroke [0.56 (0.42–0.75), *p* = 0.028, NNT = 208]. All the additional antithrombotic agents increased major bleeding except clopidogrel monotherapy in comparison with aspirin. No other significant differences were observed between the various antithrombotic treatment strategies with respect to mortality or fatal bleeding (Figure 2; Supplementary Data Sheet S2; Supplementary Figure S2).

### Subgroup studies

**FIGURE 4:**
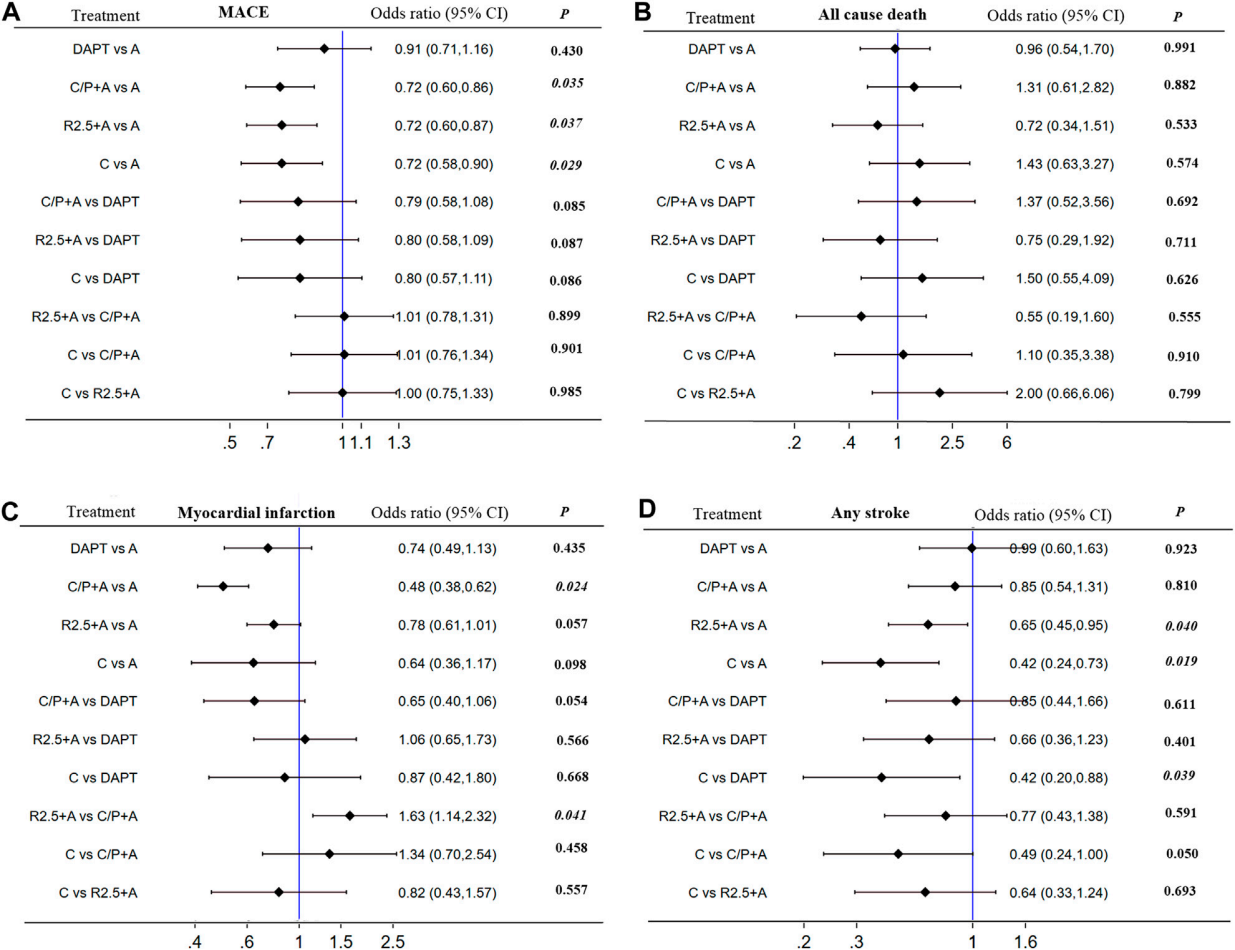
Subgroup analyses (*I_2_* = 0%) including direct and indirect comparison. MACE, all cause death, myocardial infarction and any stroke in patientswith CCS after PCI: Forest plot (estimates as hazard ratio)—All trials.

The study by Park SJ (Collet et al., 2014) was excluded because it combined the results of REAL-LATE and ZEST-LATE trials. The two studies by Bonaca MP (Bonaca et al., 2015) and Steg PG (Steg et al., 2019) were excluded as these included patients without PCI. Additionally, the COMPASS trial was replaced by COMPASS-PCI study with all included patients receiving PCI treatment (Bainey et al., 2020). Finally, 6 studies were included in this subgroup analysis with no significant heterogeneity (*I* ^*2*^ *=* 0%). In comparison with aspirin alone, addition of prasugrel to aspirin resulted in a lower risk of MACE [0.72 (0.60–0.86), *p* = 0.035, NNT = 64], MI [0.48 (0.38–0.62), *p* = 0.024, NNT = 49], and stent thrombosis [0.29 (0.09–0.91), *p* = 0.041, NNT = 106] at the expense of major bleeding [1.79 (1.34–2.39), *p* = 0.027, NNT = 84]. Similarly, addition of rivaroxaban 2.5 mg (twice daily) to aspirin was associated with a lower risk of MACE [0.72 (0.60–0.87), *p* = 0.037, NNT = 69], cardiac death [0.71 (0.52–0.98), *p* = 0.043, NNT = 189], and any stroke [0.65 (0.45–0.95), *p* = 0.040, NNT = 133], and higher risk of major bleeding [1.72 (1.33–2.22), *p* = 0.029, NNT = 71]. Clopidogrel monotherapy was associated with a lower risk of MACE [0.72 (0.58–090), *p* = 0.039, NNT = 51], any stroke [0.42 (0.24–0.73), *p* = 0.019, NNT = 109], and major bleeding [0.62 (0.40–0.96), *p* = 0.043, NNT = 139] in comparison with aspirin, but with no significant difference with respect to risk of MI. No other differences were found with respect to all-cause death, fatal bleeding, or any revascularization events (Figure 4; Supplementary Data Sheet S2; Supplementary Figure S3).

Extended aspirin with clopidogrel after 12 months showed no significant reduction in MACE, mortality, or ischemic events, but led to an increased risk of major bleeding in comparison with aspirin monotherapy in patients with CCS after PCI.

### Network coherence and quality of evidence

The risk of bias contributions of the included studies are shown in Supplementary Data Sheet S2; Supplementary Figure S6. The heterogeneity, incoherence, and report result of the mixed evidence of included studies were low, while those of the indirect evidence of the included studies were low to moderate by the CINeMA framework study (Supplementary Figures S3–S5).

## Discussion

The main findings of the present network meta-analysis were as follows: 1) adding a P2Y12 inhibitor, either prasugrel (the optimal choice) or ticagrelor was associated with a lower risk of MI; in addition, adding prasugrel was found to reduce the risk of MACE and stent thrombosis at the expense of major bleeding. 2) Clopidogrel monotherapy was found to be superior to aspirin with respect to reducing any stroke, readmission due to acute coronary syndrome (ACS) (no MI), major bleeding, and intracranial bleeding. 3) Extended-term DAPT (aspirin + clopidogrel) was not found to be superior to aspirin monotherapy in CCS after PCI. 4) Addition of low-dose anticoagulant to aspirin reduced the risk of cardiac death and any stroke, but increased the risk of major bleeding and intracranial bleeding.

CCS is defined as a group of clinical syndromes in different evolutionary stages of CAD, excluding situations with ACS (Knuuti et al., 2019). The goal of CCS therapy is to reduce cardiovascular events including MI and mortality, with a focus on reducing acute thrombotic events and the development of ventricular dysfunction. Lifelong antiplatelet therapy with aspirin has been considered as essential for the secondary prevention of MI and cardiovascular disease (CVD) in CCS patients (Knuuti et al., 2019). However, recent trials in the primary prevention setting have shown inconsistent benefits of aspirin in terms of reducing CVD events; in addition, aspirin may be associated with an increased risk of bleeding (McNeil et al., 2018). Therefore, there is no clear consensus on the optimal antithrombotic treatment strategy, including replacement of aspirin with other antiplatelet agents or addition of a P2Y12 inhibitor or a low-dose anticoagulant to aspirin for patients with CCS.

Our network meta-analysis results showed that adding a P2Y12 inhibitor, either prasugrel or ticagrelor, reduced the risk of MI, and adding prasugrel resulted in lower MACE and MI in CCS after PCI. Only the Dual Antiplatelet Therapy (DAPT) study, which included about 35% of patients receiving prasugrel (remaining 65% of patients received clopidogrel), showed a reduced risk of MACE, MI, and stent thrombosis compared with aspirin in CCS patients after PCI (Mauri et al., 2014). However, administration of aspirin in combination with clopidogrel in REAL-LATE and ZEST-LATE trial (Park et al., 2010), ARCTIC-Interruption trial (Collet et al., 2014), DES LATE trial (Lee et al., 2014), OPTIDUAL trial (Helft et al., 2016), and the mono-clopidogrel therapy of HOST-EXAM trial (Koo et al., 2021) showed no significant reduction in MI in comparison with aspirin monotherapy. Adding a P2Y12 inhibitor prasugrel may be the optimal antithrombotic strategy for patients with CCS after PCI. Compared with clopidogrel, prasugrel was shown to have greater antiplatelet efficacy in preventing thrombotic events and was unaffected by drug interactions or CYP2C19 loss-of-function (LOF) variants. Prasugrel was more effective than clopidogrel in reducing rates of ischemic events in patients with ACS after PCI (Wiviott et al., 2007), but not in medically managed patients with ACS (Roe et al., 2012), and was associated with more major bleeding events. In the HOST-REDUCEPOLYTECH-ACS study, Asian ACS patients receiving DAPT with a prasugrel de-escalation strategy (10–5 mg daily) from 1 month after PCI showed a reduced risk of (Kim et al., 2020) composite adverse clinical events [0.70 (0.52–0.92)] up to 1 year, mainly driven by a reduction in bleeding [0.48 (0.32–0.73)] without an increase in ischemia [0.76 (0.40–1.45)]. In Japanese patients with CCS after PCI, low-dose prasugrel (3.75 mg daily) achieved more consistent antiplatelet effects (P2Y12 reaction unit: 133.0 vs. 156.8, *p* = 0.005 on day 5; 124.3 vs. 158.0, *p* < 0.001 on day 30) than clopidogrel irrespective of the metabolic phenotype (Akimaru et al., 2022). Prasugrel is the most potent antiplatelet agent that can inhibit acute thrombosis in the coronary arteries; however, the concomitant risk of bleeding should also be considered. Low-dose maintenance of prasugrel may be the optimal antithrombotic strategy for CCS patients after PCI.

Ticagrelor has the predictable and consistently high level of antiplatelet effect. Compared with clopidogrel, the PLATO study showed that ticagrelor had a greater reduction in ischemic events in aspirin-treated ACS patients, but at the cost of increased risk of non-fatal bleeding (Wallentin et al., 2014). In patients with a history of MI in the preceding 1–3 years, the PEGASUS-TIMI 54 study showed that aspirin combined with ticagrelor (either 90 or 60 mg twice daily) equivalently reduced the 3-year incidence of MI, stroke, or cardiovascular death at the expense of increasing non-fatal bleeding (Bonaca et al., 2015). Similarly, the THEMIS study included CCS patients with diabetes without a history of MI or stroke; in these patients, ticagrelor (two ticagrelor doses) plus aspirin was associated with a lower incidence of MI and stroke, but a higher incidence of major bleeding compared to those who received placebo plus aspirin (Steg et al., 2019). In a recent real-world study, ticagrelor was associated with lower incidence of major adverse cardiovascular and cerebrovascular events without an increase in bleeding events in CCS after PCI in comparison with clopidogrel (Li et al., 2021). From the indirect comparison in our study, prasugrel was found superior to ticagrelor in terms of reducing MI. Ticagrelor may cause dyspnea, which occasionally necessitated switch to a thienopyridine (Storey et al., 2010) and the incidence of MI in the dyspnea group was higher than that in the no dyspnea group (112 (8.7) vs. 393 (5.4), *p* = 0.008) (Storey et al., 2011). Additionally, ticagrelor is metabolized *via* CYP3A, and therefore, should not be used with strong CYP3A inhibitors or inducers during maintenance therapy.

In the HOST-EXAM study, clopidogrel monotherapy was associated with a reduced risk of future composite of adverse clinical events (mainly stroke, readmission due to ACS and major bleeding events), but no MI during the 24-month follow-up in patients with CCS after PCI (Koo et al., 2021). We must recognize that the primary endpoint of MACE included major bleeding events, and therefore, may have confounded the statistical difference in MACE. The CAPRIE trial showed that clopidogrel is more effective than aspirin in reducing the combined risk of ischemic stroke, MI, or vascular death in patients with ACS (CAPRIE Steering Committee, 1996). In the past, clopidogrel was considerably more expensive than aspirin. Now with the expiration of patent protection, clopidogrel is considered more cost-effective, especially with the lower risk of bleeding events during long-term treatment for CCS compared to aspirin (Jones et al., 2004). From our network study, only clopidogrel monotherapy was found to reduce bleeding events among all antithrombotic drugs. Avoiding the risk of bleeding associated with antiplatelet de-escalation therapy in CCS after PCI may be more important than preventing stent thrombosis; this is particularly important in the Asian population which has a higher risk of bleeding compared to patients from Western countries. Clopidogrel is limited by poor metabolism of the hepatic cytochrome P450 enzyme CYP2C19 and the LOF variant of the *CYP2C19* gene, resulting in a lack of efficacy in some patients. Therefore, carriers of the CYP2C19 LOF allele receiving clopidogrel are at a higher risk of ischemic events compared with non-carriers (Mega et al., 2010). Finally, clopidogrel monotherapy may not be given in ACS patients from the study of STOPDAPT-2 ACS.

In comparison with aspirin alone, dual pathway inhibition with a combination of low-dose rivaroxaban (2.5 mg twice daily) and aspirin reduced the risk of stroke or cardiovascular death, but not MI; this may have been attributable to the increased risk of bleeding events in CCS, regardless of the timing (at least 1 year beyond) of prior PCI or MI in the COPASS-PCI study (Bainey et al., 2020). Similar result was not observed in ACS patients or those with prior PCI less than 1 year ago (Mega et al., 2012). Larger studies are required to substantiate this finding. In addition, the safety of performing PCI without aspirin pre-treatment is unknown.

There are several potential limitations of this meta-analysis. First, the definition of MACE and major bleeding varied among the included studies. The MACEs in the OPTIDUAL and HOST-EXAM studies included major bleeding endpoints. Second, none of the included studies had independently compared prasugrel with aspirin in CCS patients. Large RCTs are required for a more definitive assessment of the efficacy by the results of network meta-analysis. Third, the time of follow-up was different between studies. Finally, no large RCTs comparing ticagrelor (90 mg or 60 mg b.i.d) or prasugrel monotherapy with aspirin have been conducted in CCS patients after PCI.

## Conclusion

In CCS patients after PCI, adding prasugrel or ticagrelor led to a reduced incidence of MI and prasugrel was also found to reduce the risk of MACE and stent thrombosis at the expense of major bleeding. Dual pathway inhibition with a combination of low-dose rivaroxaban and aspirin reduced the risk of stroke or cardiovascular death at the cost of increased risk of bleeding. Clopidogrel monotherapy has the advantage of reducing MACE, stroke, and major bleeding events in CCS patients at high risk of bleeding after PCI. Indeed, a clinician-patient shared decision making seems apt when discussing the optimal duration of DAPT combined with the patient’s risk factors of current ischemia and bleeding.

## Data Availability

The original contributions presented in the study are included in the article/Supplementary Material, further inquiries can be directed to the corresponding authors.
