# Supplementary material for: Comparative efficacy and safety of antiplatelet or anticoagulant therapy in patients with chronic coronary syndromes after percutaneous coronary intervention: A network meta-analysis of randomized controlled trials
Source: Front Pharmacol. 2022 Sep 30;13:992376. doi: 10.3389/fphar.2022.992376 (PMC9563230; doi:10.3389/fphar.2022.992376)
Supplement: Supplementary file 1 [file DataSheet2.PDF]

Confidence In Network Meta Analysis - CINeMA 2.0.0 - Project: **Treatment in CCS network analysis**

Incoherence

Define clinically important size of effect: Odds ratio 1

*Relative effect estimates below **1.000** and above **1.000** are considered clinically important. Importance of Incoherence depends on the variability of direct and indirect effects in relation to a clinically important size of effect*

Global test based on a random-effects design-by-treatment interaction model

χ^2 **statistic: 0.000 (0 degrees of freedom), P value: NA**

Local tests: Separating indirect from direct evidence

Comparison **A:C**
Evidence: direct

Direct odds ratio: **1.382(1.113,1.716)**
Inconsistency measures: Not applicable

Incoherence judgment: No concerns

Comparison **A:C/P+A**
Evidence: direct

Direct odds ratio: **1.395(1.162,1.674)**
Inconsistency measures: Not applicable

Incoherence judgment: No concerns

Comparison **A:DAPT**
Evidence: direct

Direct odds ratio: **1.000(0.794,1.261)**
Inconsistency measures: Not applicable

Incoherence judgment: No concerns

Comparison **A:R2.5+A**
Evidence: direct

Direct odds ratio: **1.354(1.174,1.561)**
Inconsistency measures: Not applicable

Incoherence judgment: No concerns

Comparison **A:R5**
Evidence: direct

Direct odds ratio: **1.125(0.981,1.289)**
Inconsistency measures: Not applicable

Incoherence judgment: No concerns

Comparison **A:T60+A**
Evidence: direct

Direct odds ratio: **1.199(1.058,1.360)**
Inconsistency measures: Not applicable

Incoherence judgment: No concerns

Comparison **A:T90+A**
Evidence: direct

Direct odds ratio: **1.185(1.046,1.342)**
Inconsistency measures: Not applicable

Incoherence judgment: No concerns

Comparison **A:T90/60+A**
Evidence: direct

Direct odds ratio: **1.124(1.013,1.247)**
Inconsistency measures: Not applicable

Incoherence judgment: No concerns

Comparison **R2.5+A:R5**
Evidence: direct

Direct odds ratio: **0.831(0.718,0.962)**
Inconsistency measures: Not applicable

Incoherence judgment: No concerns

Comparison **T60+A:T90+A**
Evidence: direct

Direct odds ratio: **0.988(0.867,1.125)**
Inconsistency measures: Not applicable

Incoherence judgment: No concerns

Comparison **C:C/P+A**
Evidence: indirect

Indirect odds ratio: **1.010(0.761,1.340)**
Inconsistency measures: Not applicable

Incoherence judgment: No concerns

Comparison **C:DAPT**
Evidence: indirect

Indirect odds ratio: **0.724(0.527,0.994)**
Inconsistency measures: Not applicable

Incoherence judgment: Some concerns

Comparison **C:R2.5+A**
Evidence: indirect
Indirect odds ratio: **0.980(0.756,1.270)**
Inconsistency measures: Not applicable
Incoherence judgment: No concerns

Comparison **C:R5**
Evidence: indirect
Indirect odds ratio: **0.814(0.630,1.051)**
Inconsistency measures: Not applicable
Incoherence judgment: No concerns

Comparison **C:T60+A**
Evidence: indirect
Indirect odds ratio: **0.868(0.676,1.115)**
Inconsistency measures: Not applicable
Incoherence judgment: Major concerns

Comparison **C:T90+A**
Evidence: indirect
Indirect odds ratio: **0.857(0.668,1.101)**
Inconsistency measures: Not applicable
Incoherence judgment: No concerns

Comparison **C:T90/60+A**
Evidence: indirect
Indirect odds ratio: **0.813(0.640,1.034)**
Inconsistency measures: Not applicable
Incoherence judgment: No concerns

Comparison **C/P+A:DAPT**
Evidence: indirect
Indirect odds ratio: **0.717(0.534,0.963)**
Inconsistency measures: Not applicable
Incoherence judgment: Major concerns

Comparison **C/P+A:R2.5+A**
Evidence: indirect
Indirect odds ratio: **0.970(0.770,1.223)**
Inconsistency measures: Not applicable
Incoherence judgment: No concerns

Comparison **C/P+A:R5**
Evidence: indirect
Indirect odds ratio: **0.806(0.642,1.013)**
Inconsistency measures: Not applicable
Incoherence judgment: No concerns

Comparison **C/P+A:T60+A**
Evidence: indirect

Indirect odds ratio: **0.860(0.689,1.073)**
Inconsistency measures: Not applicable

Incoherence judgment No concerns

Comparison **C/P+A:T90+A**
Evidence: indirect

Indirect odds ratio: **0.849(0.681,1.059)**
Inconsistency measures: Not applicable

Incoherence judgment No concerns

Comparison **C/P+A:T90/60+A**
Evidence: indirect

Indirect odds ratio: **0.806(0.653,0.994)**
Inconsistency measures: Not applicable

Incoherence judgment No concerns

Comparison **DAPT:R2.5+A**
Evidence: indirect

Indirect odds ratio: **1.354(1.031,1.776)**
Inconsistency measures: Not applicable

Incoherence judgment Some concerns

Comparison **DAPT:R5**
Evidence: indirect

Indirect odds ratio: **1.124(0.860,1.471)**
Inconsistency measures: Not applicable

Incoherence judgment Some concerns

Comparison **DAPT:T60+A**
Evidence: indirect

Indirect odds ratio: **1.199(0.922,1.560)**
Inconsistency measures: Not applicable

Incoherence judgment Some concerns

Comparison **DAPT:T90+A**
Evidence: indirect

Indirect odds ratio: **1.184(0.911,1.541)**
Inconsistency measures: Not applicable

Incoherence judgment Some concerns

Comparison **DAPT:T90/60+A**
Evidence: indirect

Indirect odds ratio: **1.124(0.872,1.448)**
Inconsistency measures: Not applicable

Incoherence judgment Some concerns

Comparison **R2.5+A:T60+A**
Evidence: indirect

Indirect odds ratio: **0.886(0.733,1.072)**
Inconsistency measures: Not applicable

Incoherence judgment No concerns

Comparison **R2.5+A:T90+A**
Evidence: indirect

Indirect odds ratio: **0.875(0.724,1.058)**
Inconsistency measures: Not applicable

Incoherence judgment No concerns

Comparison **R2.5+A:T90/60+A**
Evidence: indirect

Indirect odds ratio: **0.830(0.696,0.991)**
Inconsistency measures: Not applicable

Incoherence judgment No concerns

Comparison **R5:T60+A**
Evidence: indirect

Indirect odds ratio: **1.067(0.886,1.284)**
Inconsistency measures: Not applicable

Incoherence judgment No concerns

Comparison **R5:T90+A**
Evidence: indirect

Indirect odds ratio: **1.053(0.875,1.268)**
Inconsistency measures: Not applicable

Incoherence judgment No concerns

Comparison **R5:T90/60+A**
Evidence: indirect

Indirect odds ratio: **1.000(0.842,1.187)**
Inconsistency measures: Not applicable

Incoherence judgment No concerns

Comparison **T60+A:T90/60+A**
Evidence: indirect

Indirect odds ratio: **0.937(0.796,1.103)**
Inconsistency measures: Not applicable

Incoherence judgment No concerns

Comparison **T90/60+A:T90+A**
Evidence: indirect

Indirect odds ratio: **1.054(0.896,1.240)**
Inconsistency measures: Not applicable

Incoherence judgment No concerns
