# Supplementary material for: Comparative efficacy and safety of antiplatelet or anticoagulant therapy in patients with chronic coronary syndromes after percutaneous coronary intervention: A network meta-analysis of randomized controlled trials
Source: Front Pharmacol. 2022 Sep 30;13:992376. doi: 10.3389/fphar.2022.992376 (PMC9563230; doi:10.3389/fphar.2022.992376)
Supplement: Supplementary file 2 [file Presentation1.pdf]

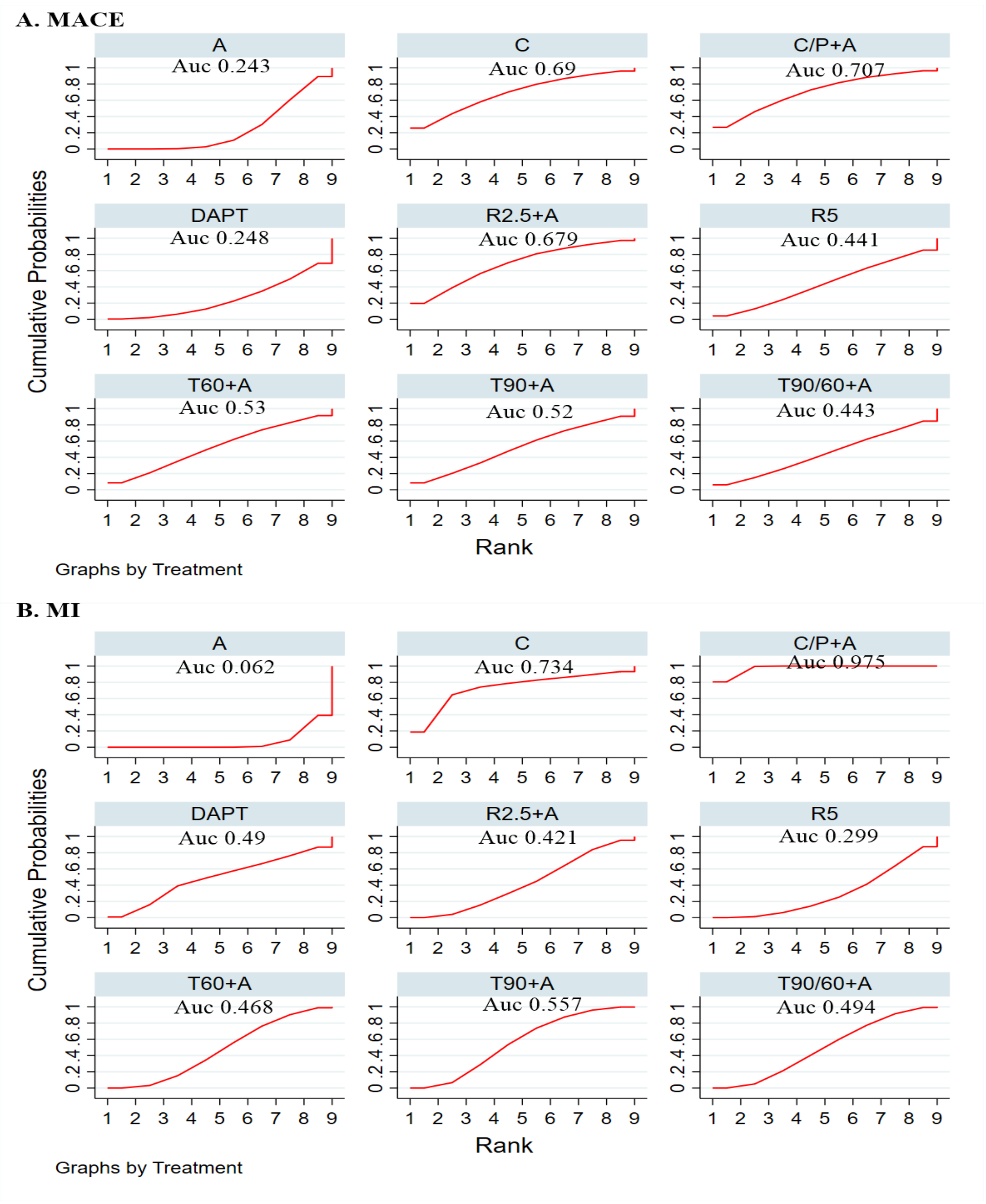

eFigure 1. Treatment strategy for MACE and MI (SUCRA rankogram plots): both the best treatment for reducing MACE and MI was prasugrel in CCS after PCI.

A=aspirin; DAPT=double antiplatelet; C/P+A=clopidogrel/prasugrel plus aspirin; T90+A=ticagrelor 90 mg twice a day plus aspirin; T60=ticagrelor 60 mg twice a day plus aspirin; T90/60+A=ticagrelor 90 mg/60 mg twice a day plus aspirin; R2.5+A=rivaroxaban 2.5 mg twice a day plus aspirin; R5=rivaroxaban 5 mg twice a day; C=clopidogrel.

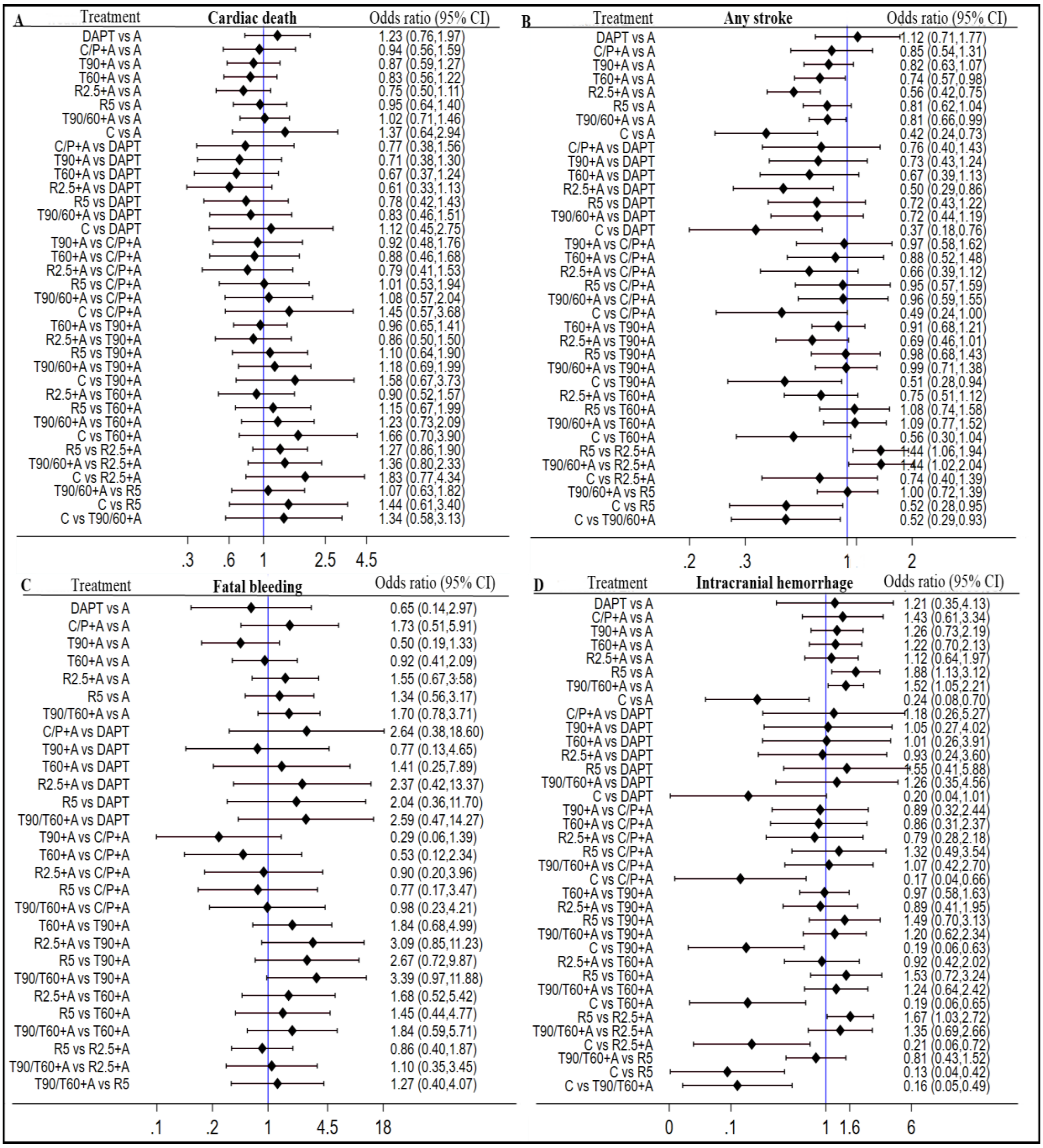

eFigure 2. Cardiac death, any stroke, fatal bleeding, and intracranial hemorrhage in patients with CCS: Forest plot (estimates as hazard ratio) - All trials

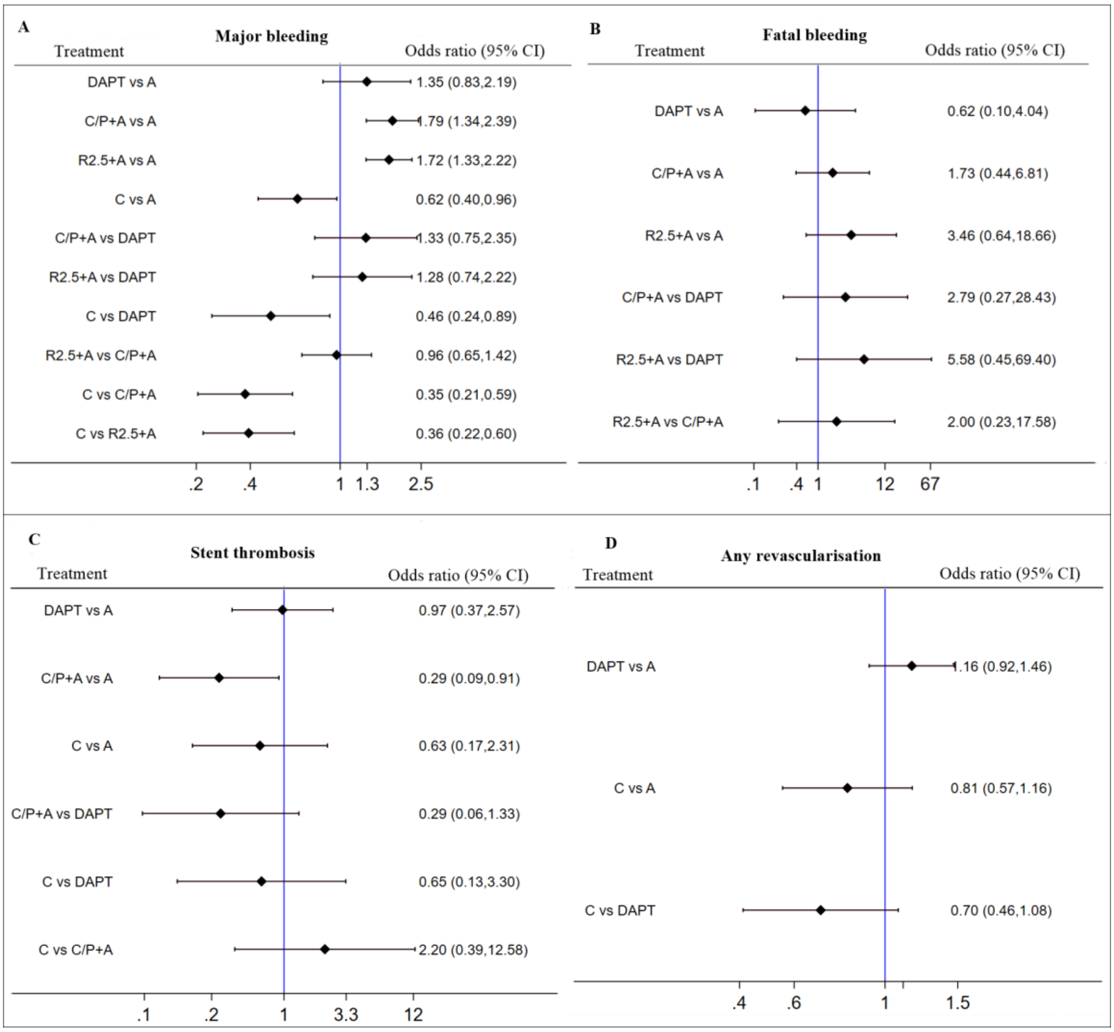

eFigure 3. Subgroup analyses including major bleeding, fatal bleeding, stent thrombosis and any revascularisation in patients with CCS after PCI: Forest plot (estimates as hazard ratio) - All trials.

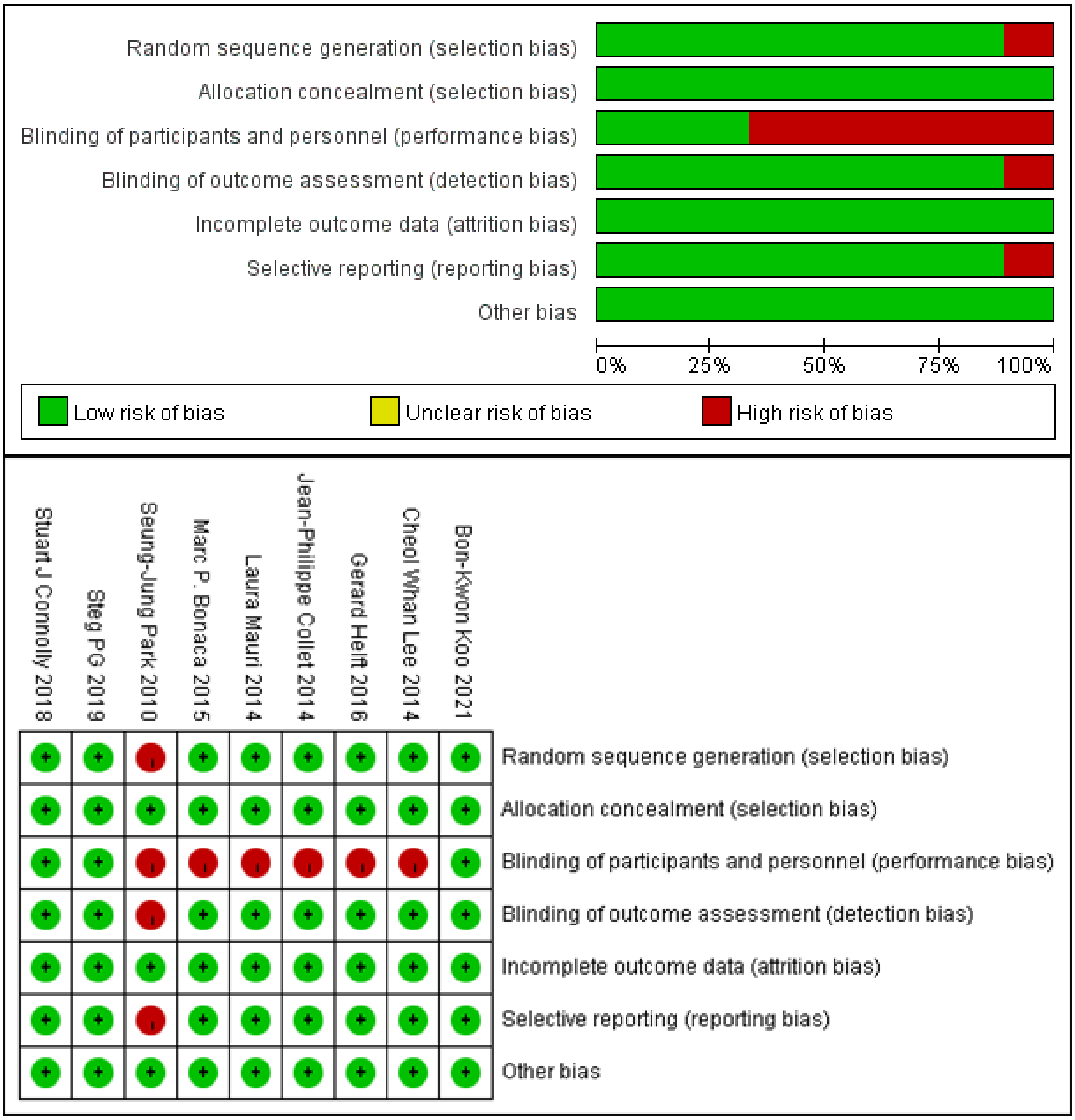

eFigure 4. Risk of bias in all trials

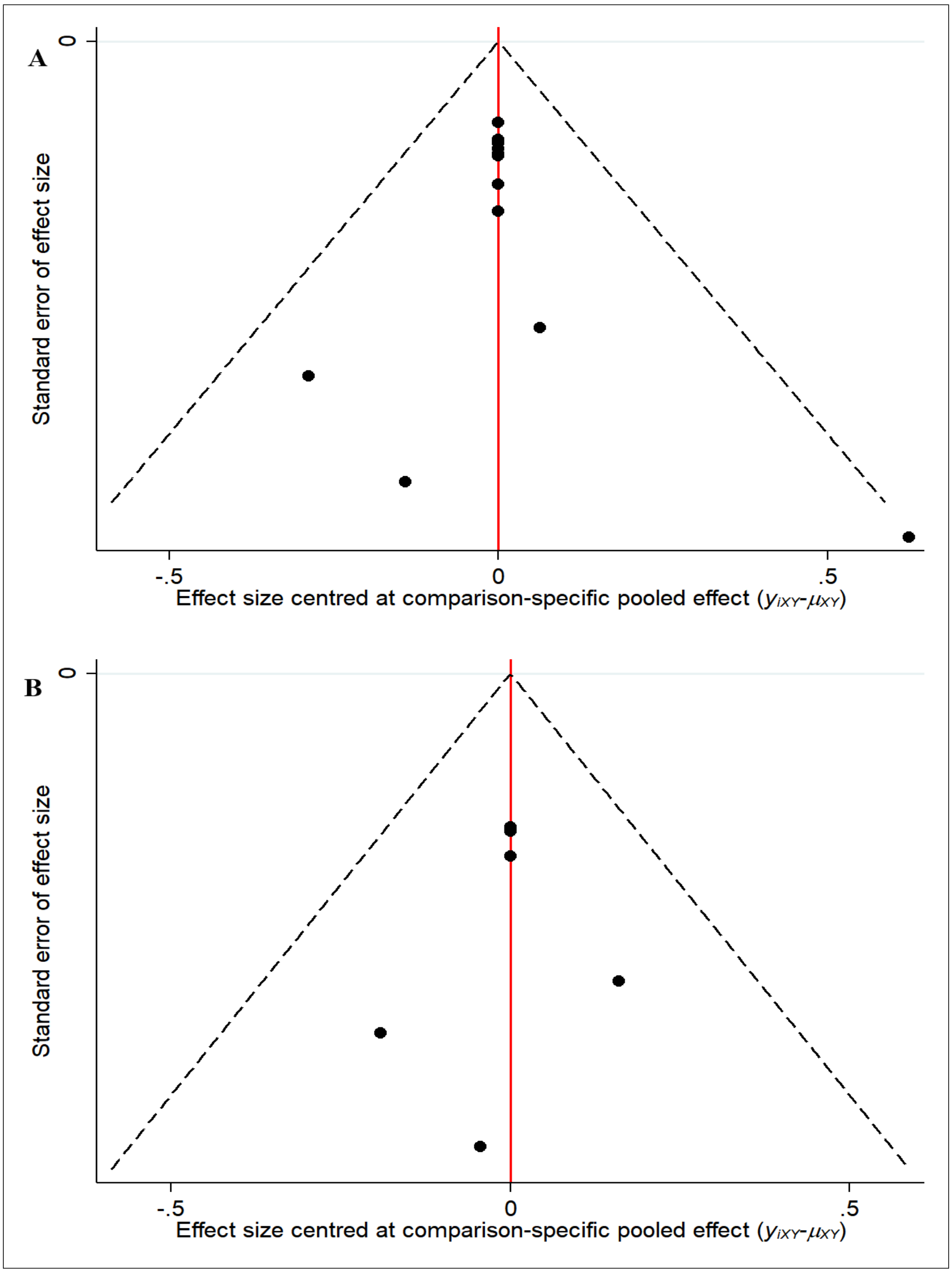

eFigure 5. Assessment of risk of bias in the included studies for MACE in patients with CCS after PCI. (A). I^2=71.9%. (B). I^2=0%..

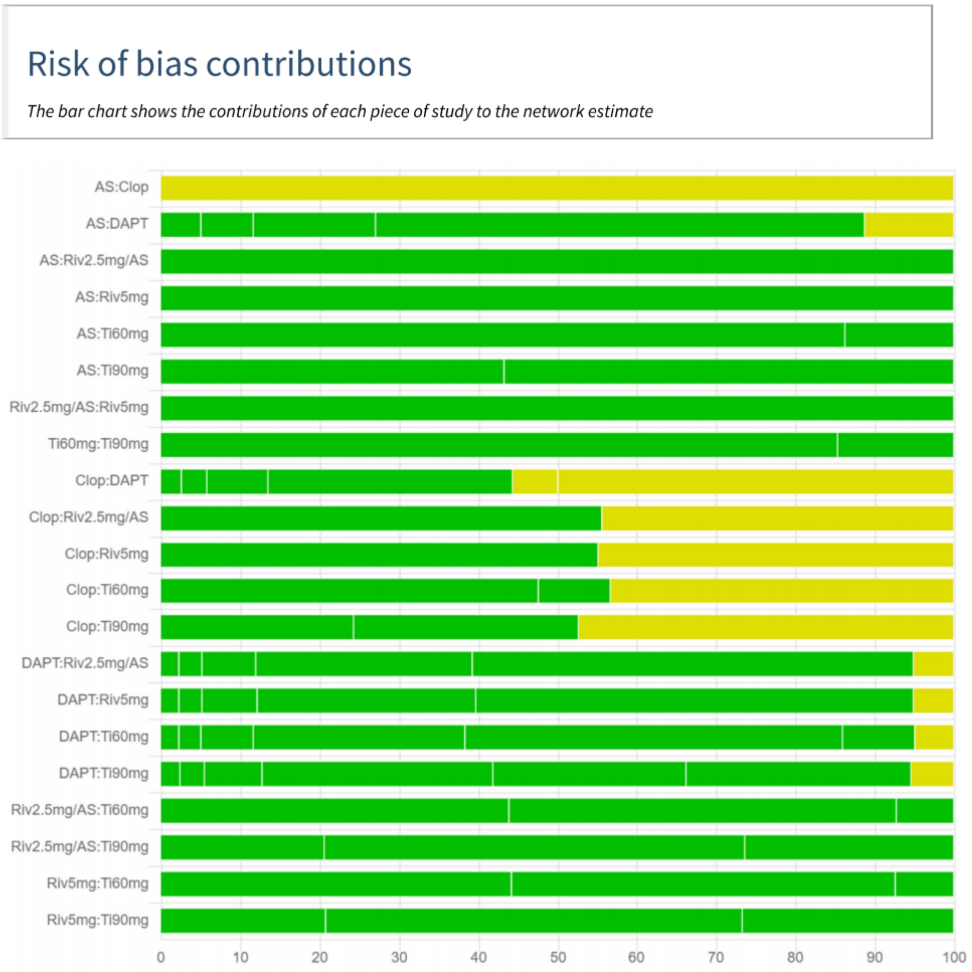

eFigure 6. The bar chart shows the contributions of each piece of study to the network estimate
