# Supplementary material for: Comparative efficacy and safety of antiplatelet or anticoagulant therapy in patients with chronic coronary syndromes after percutaneous coronary intervention: A network meta-analysis of randomized controlled trials
Source: Front Pharmacol. 2022 Sep 30;13:992376. doi: 10.3389/fphar.2022.992376 (PMC9563230; doi:10.3389/fphar.2022.992376)
Supplement: Supplementary file 3 [file DataSheet3.PDF]

Confidence In Network Meta Analysis - CINeMA 2.0.0 - Project: **Treatment in CCS network analysis**

Treatment in CCS network analysis

Comparison	Number of Studies	Within-study bias	Reporting bias	Indirectness	Imprecision	Heterogeneity	Incoherence	Confidence rating	Reason(s downgra
				Mixed evidence					
A vs C	1	No concerns	Low risk	No concerns	No concerns	No concerns	No concerns	Low	|
A vs C/P+A	1	No concerns	Low risk	No concerns	No concerns	No concerns	No concerns	Low	|
A vs DAPT	4	Some concerns	Some concerns	Some concerns	Major concerns	Some concerns	No concerns	High	|
A vs R2.5+A	1	No concerns	Low risk	No concerns	No concerns	No concerns	No concerns	Low	|
A vs R5	1	No concerns	Low risk	No concerns	No concerns	No concerns	No concerns	Low	|
A vs T60+A	1	No concerns	Low risk	No concerns	No concerns	No concerns	No concerns	Low	|
A vs T90+A	1	No concerns	Low risk	No concerns	No concerns	No concerns	No concerns	Low	|
A vs T90/60+A	1	No concerns	Low risk	No concerns	No concerns	No concerns	No concerns	Low	|
R2.5+A vs R5	1	No concerns	Low risk	No concerns	No concerns	No concerns	No concerns	Low	|
T60+A vs T90+A	1	No concerns	Low risk	No concerns	No concerns	No concerns	No concerns	Low	|
				Indirect evidence					
C vs C/P+A	--	No concerns	Low risk	No concerns	No concerns	No concerns	No concerns	Low	|
C vs DAPT	--	Some concerns	Some concerns	Major concerns	Some concerns	Some concerns	Some concerns	High	|
C vs R2.5+A	--	No concerns	Low risk	No concerns	No concerns	No concerns	No concerns	Low	|
C vs R5	--	No concerns	Low risk	No concerns	No concerns	No concerns	No concerns	Low	|
C vs T60+A	--	No concerns	Low risk	No concerns	No concerns	No concerns	Major concerns	Moderate	|
C vs T90+A	--	No concerns	Low risk	No concerns	No concerns	No concerns	No concerns	Low	|
C vs T90/60+A	--	No concerns	Low risk	No concerns	No concerns	No concerns	No concerns	Low	|

Comparison	Number of Studies	Within-study bias	Reporting bias	Indirectness	Imprecision	Heterogeneity	Incoherence	Confidence rating	Reason(s downgra
C/P+A vs DAPT	--	Some concerns	Some concerns	Some concerns	Some concerns	Some concerns	Major concerns	High	|
C/P+A vs R2.5+A	--	No concerns	Low risk	No concerns	No concerns	No concerns	No concerns	Low	|
C/P+A vs R5	--	No concerns	Low risk	No concerns	No concerns	No concerns	No concerns	Low	|
C/P+A vs T60+A	--	No concerns	Low risk	No concerns	No concerns	No concerns	No concerns	Low	|
C/P+A vs T90+A	--	No concerns	Low risk	No concerns	No concerns	No concerns	No concerns	Low	|
C/P+A vs T90/60+A	--	No concerns	Low risk	No concerns	No concerns	No concerns	No concerns	Low	|
DAPT vs R2.5+A	--	Some concerns	Some concerns	Some concerns	Some concerns	Some concerns	Some concerns	Moderate	|
DAPT vs R5	--	Some concerns	Some concerns	Some concerns	Some concerns	Some concerns	Some concerns	Moderate	|
DAPT vs T60+A	--	Some concerns	Some concerns	Some concerns	Some concerns	Some concerns	Some concerns	Moderate	|
DAPT vs T90+A	--	Some concerns	Some concerns	Some concerns	Some concerns	Some concerns	Some concerns	Moderate	|
DAPT vs T90/60+A	--	Some concerns	Some concerns	Some concerns	Some concerns	Some concerns	Some concerns	Moderate	|
R2.5+A vs T60+A	--	No concerns	Low risk	No concerns	No concerns	No concerns	No concerns	Low	|
R2.5+A vs T90+A	--	No concerns	Low risk	No concerns	No concerns	No concerns	No concerns	Low	|
R2.5+A vs T90/60+A	--	No concerns	Low risk	No concerns	No concerns	No concerns	No concerns	Low	|
R5 vs T60+A	--	No concerns	Low risk	No concerns	No concerns	No concerns	No concerns	Low	|
R5 vs T90+A	--	No concerns	Low risk	No concerns	No concerns	No concerns	No concerns	Low	|
R5 vs T90/60+A	--	No concerns	Low risk	No concerns	No concerns	No concerns	No concerns	Low	|
T60+A vs T90/60+A	--	No concerns	Low risk	No concerns	No concerns	No concerns	No concerns	Low	|
T90+A vs T90/60+A	--	No concerns	Low risk	No concerns	No concerns	No concerns	No concerns	Low	|
