# Supplementary material for: Comparative efficacy and safety of antiplatelet or anticoagulant therapy in patients with chronic coronary syndromes after percutaneous coronary intervention: A network meta-analysis of randomized controlled trials
Source: Front Pharmacol. 2022 Sep 30;13:992376. doi: 10.3389/fphar.2022.992376 (PMC9563230; doi:10.3389/fphar.2022.992376)
Supplement: Supplementary file 4 [file Table1.doc]

**eTable 1 . Final search strategy for PubMed**

| **#ID** | **Topic or intervention** | **Query** | **Records** |
| --- | --- | --- | --- |
| **#1** | Disease | (“coronary artery disease”[Mesh]) OR (“chronic coronary syndromes”[Mesh]) OR (“percutaneous coronary intervention”[Mesh]) OR (“prior myocardial infarction”[Mesh]) OR (“drug eluting stent ” [Mesh]) OR (“coronary artery disease”[Title/Abstract]) OR (“chronic coronary syndromes”[Title/Abstract]]) OR (“percutaneous coronary intervention”[Title/Abstract]) OR (“prior myocardial infarction”[Title/Abstract]) OR (“drug eluting stent ” [Title/Abstract]) | 192,392 |
| **#2** | Study design | (“randomized controlled trial”[Publication Type] OR (randomized[Publication Type]) OR “randomized controlled trial”[Title/Abstract] OR randomization [Title/Abstract] | 1,366,364 |
| **#3** | Drug strategies | (“antiplatelet therapy”[Mesh]) OR (“thienopyridines” [Mesh] ) OR (“ticagrelor” [Mesh] ) OR (“clopidogrel” [Mesh] ) OR (“aspirin” [Mesh]) OR (“prasugrel” [Mesh]) OR (“anticoagulant” [Mesh] ) OR (“[rivaroxaban](javascript:;)” [Mesh] ) OR (“apixaban” [Mesh]) OR (“dabigatran” [Mesh]) OR (“warfarin” [Mesh] ) OR (“antiplatelet therapy” [Title/Abstract]) OR (“thienopyridines” [Title/Abstract]) OR (“ticagrelor” [Title/Abstract] ) OR (“clopidogrel” [Title/Abstract]) OR (“aspirin” [Title/Abstract]) OR (“prasugrel” [Title/Abstract]) OR (“anticoagulant”[Title/Abstract]) OR (“[rivaroxaban](javascript:;)”[Title/Abstract] ) OR (“apixaban” [Title/Abstract]) OR (“dabigatran” [Title/Abstract]) OR (“warfarin” [Title/Abstract]) | 161,365 |
| **#13** | Final query | #1 AND #2 AND #3 | 3,746 |

**eTable 3. Final search strategy for Clinical Trial gov of Controlled Trials**

| **ID** | **Status** | **Query** | **Records** |
| --- | --- | --- | --- |
| **1** | Completed | (coronary artery disease OR chronic coronary syndromes) AND (randomized) AND (antiplatelet therapy OR thienopyridines OR ticagrelor OR clopidogrel OR aspirin OR prasugrel OR anticoagulant OR [rivaroxaban](javascript:;) OR apixaban OR dabigatran OR warfarin) | 98 |

**eTable 2. Final search strategy for Cochrane Central Register of Controlled Trials**

| **#ID** | **Topic or intervention** | **Query** | **Records** |
| --- | --- | --- | --- |
| **#1** | Disease | coronary artery disease OR chronic coronary syndromes OR percutaneous coronary intervention OR prior myocardial infarction OR drug eluting stent | 34,870 |
| **#2** | Study design | randomized controlled trial OR randomized | 1,247,371 |
| **#3** | Drug strategies | antiplatelet therapy OR thienopyridines OR ticagrelor OR clopidogrel OR aspirin OR prasugrel OR anticoagulant OR [rivaroxaban](javascript:;) OR apixaban OR dabigatran OR warfarin | 31,002 |
| **#13** | Final query | #1 AND #2 AND #3 | 4,757 |
