# Supplementary material for: Comparative efficacy and safety of antiplatelet or anticoagulant therapy in patients with chronic coronary syndromes after percutaneous coronary intervention: A network meta-analysis of randomized controlled trials
Source: Front Pharmacol. 2022 Sep 30;13:992376. doi: 10.3389/fphar.2022.992376 (PMC9563230; doi:10.3389/fphar.2022.992376)
Supplement: Supplementary file 5 [file DataSheet1.PDF]

Confidence In Network Meta Analysis - CINeMA 2.0.0 - Project: **Treatment in CCS network analysis**

Heterogeneity

Define clinically important size of effect: Odds ratio 1

*Relative effect estimates below **1.000** and above **1.000** are considered clinically important.*
Importance of heterogeneity depends on the variability of effects in relation to a clinically important size of effect

The estimated value of between-study variance for the network meta-analysis is **0.057**

17 comparisons were manually updated

Comparison **A:C**
Evidence: mixed
NMA estimate: **1.382**
95% intervals for NMA estimate
Confidence interval: **(1.113,1.716)**
Prediction interval: **(0.301,6.344)**
*Prediction interval extends into clinically important effects in **both** directions*
Heterogeneity judgment
No concerns

Comparison **A:C/P+A**
Evidence: mixed
NMA estimate: **1.395**
95% intervals for NMA estimate
Confidence interval: **(1.162,1.674)**
Prediction interval: **(0.310,6.268)**
*Prediction interval extends into clinically important effects in **both** directions*
Heterogeneity judgment
No concerns

Comparison **A:DAPT**
Evidence: mixed
NMA estimate: **1.000**
95% intervals for NMA estimate
Confidence interval: **(0.794,1.261)**
Prediction interval: **(0.277,3.453)**
*Confidence and prediction intervals **agree** in relation to clinically important effect*
Heterogeneity judgment
Some concerns

Comparison **A:R2.5+A**
Evidence: mixed
NMA estimate: **1.354**
95% intervals for NMA estimate
Confidence interval: **(1.174,1.561)**
Prediction interval: **(0.308,5.957)**
*Prediction interval extends into clinically important effects in **both** directions*
Heterogeneity judgment
No concerns

Comparison **A:R5**
Evidence: mixed

NMA estimate: **1.125**
95% intervals for NMA estimate
Confidence interval: **(0.981,1.289)**
Prediction interval: **(0.256,4.935)**

*Confidence and prediction intervals **agree** in relation to clinically important effect*

Heterogeneity judgment
No concerns

Comparison **A:T60+A**
Evidence: mixed

NMA estimate: **1.199**
95% intervals for NMA estimate
Confidence interval: **(1.058,1.360)**
Prediction interval: **(0.275,5.238)**

*Prediction interval extends into clinically important effects in **both** directions*

Heterogeneity judgment
No concerns

Comparison **A:T90+A**
Evidence: mixed

NMA estimate: **1.185**
95% intervals for NMA estimate
Confidence interval: **(1.046,1.342)**
Prediction interval: **(0.271,5.173)**

*Prediction interval extends into clinically important effects in **both** directions*

Heterogeneity judgment
No concerns

Comparison **A:T90/60+A**
Evidence: mixed

NMA estimate: **1.124**
95% intervals for NMA estimate
Confidence interval: **(1.013,1.247)**
Prediction interval: **(0.259,4.869)**

*Prediction interval extends into clinically important effects in **both** directions*

Heterogeneity judgment
No concerns

Comparison **R2.5+A:R5**
Evidence: mixed

NMA estimate: **0.831**
95% intervals for NMA estimate
Confidence interval: **(0.718,0.962)**
Prediction interval: **(0.188,3.662)**

*Prediction interval extends into clinically important effects in **both** directions*

Heterogeneity judgment
No concerns

Comparison **T60+A:T90+A**
Evidence: mixed

NMA estimate: **0.988**
95% intervals for NMA estimate
Confidence interval: **(0.867,1.125)**
Prediction interval: **(0.226,4.321)**

*Confidence and prediction intervals **agree** in relation to clinically important effect*

Heterogeneity judgment
No concerns

Comparison **C:C/P+A**
Evidence: indirect

NMA estimate: **1.010**
95% intervals for NMA estimate
Confidence interval: **(0.761,1.340)**
Prediction interval: **(0.154,6.612)**

Confidence and prediction intervals ***agree*** *in relation to clinically important effect*

Heterogeneity judgment
No concerns

Comparison **C:DAPT**
Evidence: indirect

NMA estimate: **0.724**
95% intervals for NMA estimate
Confidence interval: **(0.527,0.994)**
Prediction interval: **(0.130,3.846)**

Prediction interval extends into clinically important effects in ***both*** *directions*

Heterogeneity judgment
Some concerns

Comparison **C:R2.5+A**
Evidence: indirect

NMA estimate: **0.980**
95% intervals for NMA estimate
Confidence interval: **(0.756,1.270)**
Prediction interval: **(0.152,6.310)**

Confidence and prediction intervals ***agree*** *in relation to clinically important effect*

Heterogeneity judgment
No concerns

Comparison **C:R5**
Evidence: indirect

NMA estimate: **0.814**
95% intervals for NMA estimate
Confidence interval: **(0.630,1.051)**
Prediction interval: **(0.127,5.231)**

Confidence and prediction intervals ***agree*** *in relation to clinically important effect*

Heterogeneity judgment
No concerns

Comparison **C:T60+A**
Evidence: indirect

NMA estimate: **0.868**
95% intervals for NMA estimate
Confidence interval: **(0.676,1.115)**
Prediction interval: **(0.136,5.558)**

Confidence and prediction intervals ***agree*** *in relation to clinically important effect*

Heterogeneity judgment
No concerns

Comparison **C:T90+A**
Evidence: indirect

NMA estimate: **0.857**
95% intervals for NMA estimate
Confidence interval: **(0.668,1.101)**
Prediction interval: **(0.134,5.488)**

Confidence and prediction intervals ***agree*** *in relation to clinically important effect*

Heterogeneity judgment
No concerns

Comparison **C:T90/60+A**
Evidence: indirect

NMA estimate: **0.813**
95% intervals for NMA estimate
Confidence interval: **(0.640,1.034)**
Prediction interval: **(0.128,5.175)**

Confidence and prediction intervals ***agree*** *in relation to clinically important effect*

Heterogeneity judgment
No concerns

Comparison **C/P+A:DAPT**
Evidence: indirect

NMA estimate: **0.717**
95% intervals for NMA estimate
Confidence interval: **(0.534,0.963)**
Prediction interval: **(0.131,3.736)**

Prediction interval extends into clinically important effects in ***both*** *directions*

Heterogeneity judgment
Some concerns

Comparison **C/P+A:R2.5+A**
Evidence: indirect

NMA estimate: **0.970**
95% intervals for NMA estimate
Confidence interval: **(0.770,1.223)**
Prediction interval: **(0.153,6.141)**

Confidence and prediction intervals ***agree*** *in relation to clinically important effect*

Heterogeneity judgment
No concerns

Comparison **C/P+A:R5**
Evidence: indirect

NMA estimate: **0.806**
95% intervals for NMA estimate
Confidence interval: **(0.642,1.013)**
Prediction interval: **(0.128,5.091)**

Confidence and prediction intervals ***agree*** *in relation to clinically important effect*

Heterogeneity judgment
No concerns

Comparison **C/P+A:T60+A**
Evidence: indirect

NMA estimate: **0.860**
95% intervals for NMA estimate
Confidence interval: **(0.689,1.073)**
Prediction interval: **(0.137,5.408)**

Confidence and prediction intervals ***agree*** *in relation to clinically important effect*

Heterogeneity judgment
No concerns

Comparison **C/P+A:T90+A**
Evidence: indirect

NMA estimate: **0.849**
95% intervals for NMA estimate
Confidence interval: **(0.681,1.059)**
Prediction interval: **(0.135,5.341)**

Confidence and prediction intervals ***agree*** *in relation to clinically important effect*

Heterogeneity judgment
No concerns

Comparison **C/P+A:T90/60+A**
Evidence: indirect

NMA estimate: **0.806**

95% intervals for NMA estimate

Confidence interval: **(0.653,0.994)**

Prediction interval: **(0.129,5.035)**

*Prediction interval extends into clinically important effects in **both** directions*

Heterogeneity judgment

No concerns

Comparison **DAPT:R2.5+A**
Evidence: indirect

NMA estimate: **1.354**

95% intervals for NMA estimate

Confidence interval: **(1.031,1.776)**

Prediction interval: **(0.265,7.250)**

*Prediction interval extends into clinically important effects in **both** directions*

Heterogeneity judgment

Some concerns

Comparison **DAPT:R5**
Evidence: indirect

NMA estimate: **1.124**

95% intervals for NMA estimate

Confidence interval: **(0.860,1.471)**

Prediction interval: **(0.220,6.009)**

*Confidence and prediction intervals **agree** in relation to clinically important effect*

Heterogeneity judgment

Some concerns

Comparison **DAPT:T60+A**
Evidence: indirect

NMA estimate: **1.199**

95% intervals for NMA estimate

Confidence interval: **(0.922,1.560)**

Prediction interval: **(0.236,6.380)**

*Confidence and prediction intervals **agree** in relation to clinically important effect*

Heterogeneity judgment

Some concerns

Comparison **DAPT:T90+A**
Evidence: indirect

NMA estimate: **1.184**

95% intervals for NMA estimate

Confidence interval: **(0.911,1.541)**

Prediction interval: **(0.233,6.301)**

*Confidence and prediction intervals **agree** in relation to clinically important effect*

Heterogeneity judgment

Some concerns

Comparison **DAPT:T90/60+A**
Evidence: indirect

NMA estimate: **1.124**

95% intervals for NMA estimate

Confidence interval: **(0.872,1.448)**

Prediction interval: **(0.223,5.936)**

*Confidence and prediction intervals **agree** in relation to clinically important effect*

Heterogeneity judgment

Some concerns

Comparison **R2.5+A:T60+A**
Evidence: indirect

NMA estimate: **0.886**
95% intervals for NMA estimate
Confidence interval: **(0.733,1.072)**
Prediction interval: **(0.143,5.480)**

*Confidence and prediction intervals **agree** in relation to clinically important effect*

Heterogeneity judgment
No concerns

Comparison **R2.5+A:T90+A**
Evidence: indirect

NMA estimate: **0.875**
95% intervals for NMA estimate
Confidence interval: **(0.724,1.058)**
Prediction interval: **(0.142,5.411)**

*Confidence and prediction intervals **agree** in relation to clinically important effect*

Heterogeneity judgment
No concerns

Comparison **R2.5+A:T90/60+A**
Evidence: indirect

NMA estimate: **0.830**
95% intervals for NMA estimate
Confidence interval: **(0.696,0.991)**
Prediction interval: **(0.135,5.102)**

*Prediction interval extends into clinically important effects in **both** directions*

Heterogeneity judgment
No concerns

Comparison **R5:T60+A**
Evidence: indirect

NMA estimate: **1.067**
95% intervals for NMA estimate
Confidence interval: **(0.886,1.284)**
Prediction interval: **(0.173,6.580)**

*Confidence and prediction intervals **agree** in relation to clinically important effect*

Heterogeneity judgment
No concerns

Comparison **R5:T90+A**
Evidence: indirect

NMA estimate: **1.053**
95% intervals for NMA estimate
Confidence interval: **(0.875,1.268)**
Prediction interval: **(0.171,6.499)**

Confidence and prediction intervals ***agree*** *in relation to clinically important effect*

Heterogeneity judgment
No concerns

Comparison **R5:T90/60+A**
Evidence: indirect

NMA estimate: **1.000**
95% intervals for NMA estimate
Confidence interval: **(0.842,1.187)**
Prediction interval: **(0.163,6.127)**

Confidence and prediction intervals ***agree*** *in relation to clinically important effect*

Heterogeneity judgment
No concerns

Comparison **T60+A:T90/60+A**
Evidence: indirect

NMA estimate: **0.937**
95% intervals for NMA estimate
Confidence interval: **(0.796,1.103)**
Prediction interval: **(0.153,5.722)**

Confidence and prediction intervals ***agree*** *in relation to clinically important effect*

Heterogeneity judgment
No concerns

Comparison **T90/60+A:T90+A**
Evidence: indirect

NMA estimate: **1.054**
95% intervals for NMA estimate
Confidence interval: **(0.896,1.240)**
Prediction interval: **(0.173,6.435)**

Confidence and prediction intervals ***agree*** *in relation to clinically important effect*

Heterogeneity judgment
No concerns
